# *Candida albicans*-induced activation of the TGF-β/Smad pathway and upregulation of IL-6 may contribute to intrauterine adhesion

**DOI:** 10.1038/s41598-022-25471-0

**Published:** 2023-01-11

**Authors:** Xingping Zhao, Dan Sun, Aiqian Zhang, Huan Huang, Yueran Li, Dabao Xu

**Affiliations:** 1 Department of Gynecology, Third Xiangya Hospital of Central South University, 138 Tongzipo Road, Changsha, 410013 Hunan China; 2 The First Affiliated Hospital of Guangxi Medical University, Nanning, 530021 China

**Keywords:** Microbiology, Medical research, Pathogenesis, Risk factors

## Abstract

Iatrogenic injury to endometrial tissue is the main cause of intrauterine adhesions (IUA) and infection can also damage the endometrium. The microbiota plays an important role in the health of the female reproductive tract. However, the mechanism is still unclear. In total, 908 patients with IUA and 11,389 healthy individuals were retrospectively selected for this clinical study. Participant information including vaginal microecological results and human papillomavirus (HPV) status were collected. Univariate and multivariate logistic regression analyses were used to identify the factors related to IUA. Next, animal experiments were performed in a curettage-induced IUA rat model. After the procedure, rats in the experimental group received a vaginal infusion of a *Candida albicans* (*C. albicans*) fungal solution. On days 3, 7, and 14 after curettage and infusion, the expression levels of IL-6, fibrotic pathway-related factors (TGF-β1, Smad 2, and COL1), and estrogen receptor (ER) and progesterone receptor (PR) in rat endometrial tissues were assessed. Fungal infection of the reproductive tract was found to be an independent risk factor for IUA (*P* < 0.05). The inflammatory response and degree of fibrosis were greater in rats infected with *C. albicans* than in the controls. The levels of IL-6, TGF-β1, Smad 2, and COL1 expression in endometrial tissues were significantly higher in the experimental group than in the control group (*P* < 0.05). However, the ER and PR levels were lower in the IUA group than in the non-IUA group (*P* < 0.05). *C. albicans* infection may be related to IUA. *C. albicans* elicits a strong inflammatory response that can lead to more severe endometrial fibrosis.

## Introduction

Intrauterine adhesions (IUA) have many causes, the most common of which is iatrogenic endometrial injury^1^. Typical clinical manifestations of IUA are decreased menstruation, amenorrhea, recurrent miscarriage, and secondary infertility^2^. A common clinical observation is that some patients develop IUA after uterine cavity surgery, whereas others do not, and that the adhesions range from severe to very mild. These differences suggest that a single iatrogenic injury does not constitute the entire mechanism behind the occurrence and development of IUA, and that other pathogenic factors may be involved.

Many types of microorganisms can be found in the reproductive tracts of healthy women. A fine and balanced relationship exists between the host and microorganisms, which is conducive to maintaining the steady state of the microbiota and resisting invasion by pathogenic microorganisms. The microbiome plays an important role in disease and the health of the female reproductive tract^3,4^. Studies have confirmed that the main cause of IUA is injury to the basal layer of the endometrium, and that any event resulting in such injury can potentially lead to IUA^1^. However, in the clinical setting, patients with IUA rarely exhibit typical signs of reproductive tract infection. The only definite infection-related cause of IUA is genital tuberculosis^5^, and the etiological role of other uterine inflammatory infections in the formation of IUA remains contentious^6^.

In recent decades, the incidence of human fungal infections has increased significantly^7^. Many factors influence the occurrence of fungal diseases, including receipt of immunosuppressive therapy, invasive surgery, or broad-spectrum antibiotics. Reproductive tract fungal infections can have serious effects on women's professional and personal lives^8,9^. Harnessing 16S and ITS2 rDNA sequencing analysis, our previous study found that the fungal genera were enriched in the lower genital tract of IUA patients^10^. Moreover, site-specific fungal-bacterial correlation networks were discovered in those patients^10^. Vaginal candidiasis is the most common vaginal fungal infection^11^. The main pathogenic agent of vaginal candidiasis is the polymorphic fungal pathogen *Candida albicans* (*C. albicans*)^12^. Only 30% of recurrent vulvovaginal candidiasis cases are caused by non-*C. albicans* infections^13^.

The specific host immune response to *C. albicans* is generally considered to play a key role in protection by preventing symbiotic *Candida* from turning into conditional pathogens at the mucus membrane–*Candida* interface. Transforming growth factor (TGF)-β may be involved in the immune response to fungal infection in the reproductive tract. Taylor et al.^14^ found that TGF-β1 expression was significantly increased in the vaginal tissue of mice with candidiasis. Furthermore, TGF-β1 was found to be dominant in draining lymph nodes, but not in non-draining lymph nodes. These findings suggest that TGF-β1 may be involved in the immune regulation of vaginal *Candida* infections^14^. Other studies have found that TGF-β plays a specific role in the process of fungal morphology recognition by dendritic cells^15^. Drug therapy has been reported to downregulate the ratio of proinflammatory cytokine interleukin (IL)-1β to anti-inflammatory cytokine TGF-β, thereby reducing the number of neutrophils in the vagina^16^. TGF-β is a key cytokine in human tissue and animal models of fibrotic diseases, such as IUA^17–19^. It has been shown to have a significant effect on fibroblast proliferation^20,21^ and extracellular matrix production^22,23^ in vitro. The interplay between Smad proteins and other proteins in TGF-β signaling pathway mediate regulatory signals that control expression of target genes^24^. So they are mediators and essential components of TGF-β signaling pathway. We speculated that *C. albicans* infection may lead to IUA and that the immune regulation mechanism may be related to TGF-β/Smad signaling pathway activation by *C. albicans*.

As a fibrosis-promoting inflammatory cytokine^25^, IL-6 has been detected at significantly increased levels in patients with pulmonary fibrosis^26^, as well as in a fibrosis mouse model^27^. Recent studies have shown that IL-6 can promote fibrosis by mediating chronic inflammation^28^ and activating the TGF-β/Smad pathway^29–33^. Studies have further suggested that Smad 2/3 may be the target of IL-6 trans-signal transduction^29,34,35^. The present study took IL-6 as a starting point for further exploration of the mechanism by which *C. albicans* activates the TGF-β signal transduction pathway.

We speculated that some patients with IUA who have "asymptomatic" genital tract infections may have abnormal changes in reproductive tract microorganisms and that this vaginal microecological imbalance may be involved in the formation of fibrosis after endometrial injury. Furthermore, we posited that the imbalance of immune regulation caused by *C. albicans* infection may be an important link in the mechanism of IUA and that IL-6 may be a key regulatory factor. In this research we first retrospectively analyzed the relationship between vaginal microecology and IUA, and preliminarily investigated the etiology of IUA using logistic regression analysis. To further explore the mechanism of endometrial injury caused by fungi, *C. albicans* was used to stimulate primary intimal cells in a pregnant rat uterine curettage model, and the levels of IL-6, TGF-β1, Smad 2, collagen 1 (COLl), estrogen receptor (ER), and progesterone receptor (PR) were subsequently evaluated. By exploring the mechanism underlying *C. albicans*-mediated IL-6 upregulation in the reproductive tract and its role in IUA formation, we aimed to reveal the various roles of *C. albicans* in the progression of IUA and provide new ideas and targets for the discovery of effective preventative measures and treatments for this condition.

## Methods

### Study population

In this study, 908 patients with IUA who were diagnosed in the Gynecology Clinic of Xiangya Third Hospital of Central South University between August 2017 and September 2018 were retrospectively enrolled as the study group. A further 11,389 healthy people who attended the Health Management Center of Xiangya Third Hospital of Central South University during the same period were retrospectively selected as the control group. The clinical data of the study participants were carefully recorded and reviewed by two research assistants.

All 12,297 study participants provided written informed consent, and the study was approved by the Ethics Committee of Xiangya Third Hospital of Central South University (ethics no. I 22031). All methods were performed in accordance with the relevant guidelines and regulations of the Ethics Committee.

The inclusion criteria for participants were as follows: (i) had not used drugs or other means to treat vaginitis in the 3 months before sampling; (ii) had not engaged in sexual intercourse or other vaginal operations in the 1 day before sampling; (iii) had the ability to understand the advantages and disadvantages of participating in this study and willingness to cooperate with the sampling and testing work required; and (iv) had provided written informed consent. Participants were excluded from the study if they had not been sexually active before, had symptoms or signs of reproductive tract infection, were pregnant or lactating, were unable to cooperate with examinations, or withdrew from the study.

### Collection of clinical samples

Samples of vaginal secretions were obtained by gynecologists trained to collect secretions from the upper third of the vaginal sidewall. The swabs with vaginal secretions were inserted into aseptic test tubes and sealed. Then, the cervical mucus was wiped away with a large cotton swab, and a human papillomavirus (HPV) sampling brush was extended into the cervical tube and gently rotated clockwise five times. The front segment of the HPV sampling brush was then snapped off and stored in preservation solution. A test barcode was affixed to the specimen bottle, which was then immediately sent to the laboratory.

### Testing of vaginal secretions

The pH of vaginal secretions was detected using pH 3.8–5.4 precision pH test paper, as described previously^36^. The cleanliness of vaginal secretions was checked directly under a high-power microscope using the saltwater glass film method^37^. Samples were stained with Gram staining solution (Baso Diagnostics, Guangdong, China) following standard steps. Nugent scores were determined based on the bacterial morphology of *Lactobacillus*, *Campylobacter*, *Gardnerella*, and *Bacteroides*. Fungal infection was evaluated by observing fungal hyphae, budding spores, and spores. A vaginitis detection kit (Jiangsu Bioperfectus Technologies, Jiangsu, China) was used to evaluate the vaginal ecology. All tests on vaginal secretions were performed by experienced technicians.

### HPV test

HPV DNA was amplified by polymerase chain reaction (PCR) and detected using an HPV gene chip detection kit (HybriBio, Guangdong, China). Twenty-one types of HPV were detected, of which 14 were high risk (16, 18, 31, 33, 35, 39, 45, 51, 52, 56, 58, 59, 66, and 68), 5 were low risk (6, 11, 42, 43 and 44), and 2 were of unknown risk (53 and CP8304)^38^. If any of these HPV types were detected, a sample was considered HPV positive,if none of these HPV types were detected, a sample was considered HPV negative.

### Diagnostic information

Vaginal secretions with a pH of < 4.5 were regarded as normal, whereas those with a pH of ≥ 4.5 were regarded as abnormal^36^. Bacteria observed in the highest numbers under the microscope were defined as the dominant bacteria. Bacteria were divided into two groups: Gram-positive bacteria and Gram-negative bacteria (2016). The cleanliness of vaginal secretions was graded into four categories: grade I, mainly vaginal bacilli, with a large number of epithelial cells,grade II, some vaginal bacilli, epithelial cells, white blood cells, and miscellaneous bacteria; grade III, only a small amount of vaginal bacilli and epithelial cells, but a large number of white blood cells and miscellaneous bacteria; and grade IV, full of white blood cells and miscellaneous bacteria, with no vaginal bacilli. Grades I and II were considered normal, whereas grades III to IV was considered abnormal (2016). The Nugent score of vaginal secretions was calculated according to the Gram staining score standard^39^. Samples were considered positive for fungi if spores, budding spores, or hyphae were observed under the microscope.

### Animals

A total of 12 female Sprague Dawley (SD) rats (13–15 days pregnant, weight 400–450 g) were obtained from the Hunan SJA Laboratory Animal Co. Ltd (Hunan, China). The rats were housed in a standard environment under a 12-h light–dark cycle with 60–70% relative humidity at 22–24 °C. All animal experiments were approved by the Ethics Committee for Animal Research of Central South University and strictly complied with the guidelines and requirements of the National Institutes of Health (NIH) for the care and use of laboratory animals (Animal Ethics Committee Review no. 2020sydw0713). The study is reported in accordance with ARRIVE guidelines (Animal Research: Reporting of In Vivo Experiments).

### Reagents

A Masson’s trichrome stain kit was purchased from Solarbio (Shanghai, China). Neutral balsam, a hematoxylin and eosin (H&E) staining system, and a rabbit anti-sheep immunohistochemistry (IHC) kit were obtained from Auragene (Hunan, China). Rat TGF-β1 and rat IL-6 enzyme-linked immunosorbent assay (ELISA) kits were purchased from Shanghai Enzyme-linked Biotechnology (Shanghai, China). Primary anti-IL-6, anti-TGF-β1, anti-Smad 2, anti-COLl, anti-ER, and anti-PR antibodies, and secondary goat anti-rabbit antibodies were purchased from Abcam (Cambridge, MA, USA). *C. albicans* was obtained from the Institute of Microbiology, Chinese Academy of Sciences (Beijing, China), and cultured according to the instructions.

### Establishing an IUA model in pregnant rats

The rats were purchased at 13 days of gestation, and the experiment was carried out after 2–3 days of adaptive feeding in the laboratory. The rats were anesthetized by intraperitoneal injection of 40 mg/kg of 2% pentobarbital sodium. A median straight incision was made in the lower abdomen, and a 2–3 cm incision was made through the skin, subcutaneous tissue, rectus abdominis, and peritoneum to enter the abdominal cavity. The Y-shaped uterus was located and gently removed from the abdominal cavity and spread on the surgical hole towel. A 0.5-cm longitudinal incision was made at the proximal end of the bilateral uterus; the fetus, placenta, and other gestational tissues were removed in turn. Curettage was performed using a custom-made curette. The length of the curettage was approximately 5–10 cm from the incision. The strength of the curette was tailored to allow a large amount of tissue to be scraped out and the uterine cavity to become rough, while avoiding penetration of the uterus. The uterus was then sutured, and the abdomen closed with 5-0 silk thread.

### *C. albicans* infection assay

On day 1 after the procedure, the rats were randomly divided into two groups: the experimental group and the control group (n = 6 in each group). In the experimental group, 50 μL *C. albicans* culture fluid (with a fungal content of ~ 3 × 10^7^) was injected into the vagina of each rat using a gastric perfusion needle inserted approximately 2 cm inside the vagina (approximately at the junction of the cervical canal and the uterus). Then the *C. albicans* migrated from the vagina to the uterine cavity. In the control group, 50 μL phosphate-buffered saline (PBS) solution was administered into the vagina of each rat. There was no difference in the timing of administration between the two groups. Uterine samples were obtained from rats in each group on postoperative days 3, 7, and 14. Specimens were stored in a refrigerator at 4 °C for 24 h, then frozen at –80 °C until use.

For histological analysis, specimens were stained with H&E and Masson’s trichrome. IHC was used to detect the levels of IL-6, TGF-β1, Smad 2, COLl, ER, and PR in the endometrium, and the levels of IL-6 and TGF-β1 were measured by ELISA.

### Histological examination with H&E staining

For H&E staining, three uterus sections were randomly selected from each rat. The samples were first dewaxed and hydrated and then stained with hematoxylin for 10 min. Next, the samples were rinsed under running water for 2 min, differentiated in 1% saline alcohol for 2 s, rinsed again for 15 min, washed with distilled water for 1–2 s, and then stained with eosin for 10 min. The samples were next differentiated in an 80% ethanol solution according to their color before being dehydrated, first with 85% ethanol for 5 min, then with 95% ethanol for 5 min, and finally with anhydrous ethanol for 10 min, twice. Xylene was used to achieve transparency. After sealing with neutral gum, morphological changes in the endometrium were observed under an inverted microscope (40×).

### Masson’s trichrome staining

Samples were first dewaxed and hydrated, and then stained with a pre-prepared Weigert’s hematoxylin staining solution for 8 min. Next, the samples were differentiated with 1% acidic ethanol differentiation buffer for 10 s, before being washed with water and transferred to Masson’s blue solution for 5 min to be blued. After that, they were dyed with Masson’s magenta dye for 5 min, washed for 1 min, and then transferred directly to aniline blue dye for 2 min. Finally, the specimens were dehydrated, rendered transparent, and sealed in neutral resins.

### IHC staining

Samples were dewaxed and hydrated. After high-temperature and high-pressure antigen repair, the samples were incubated with 3% H_2_O_2_ at room temperature for 10 min to block endogenous peroxidase activity. Then, primary antibodies for IL-6, TGF-β1, Smad 2, COIL1, ER, and PR were added at a dilution of 1:200, and the samples were incubated at 4 °C overnight. PBS was used as the negative control solution. Following overnight incubation in a wet box, the samples were rewarmed to 37 °C for 30 min. Horseradish peroxidase-labeled polymer (ready-to-use; P009IH; Auragene, Hunan, China) was added to the samples, which were subsequently subjected to incubation at room temperature for 30 min, followed by washing with PBS.

Four high-power visual fields were selected in each section, and there were no blank areas in any field. Images of the IHC-stained endometrial sections were captured. Brown areas or granules in the endometrium were taken as positive. The integrated optical density (IOD) of each image was measured using Image-ProPlus 6.0 image analysis software (Media Cybernetics, Bethesda, MD, USA). The IOD values of the endometrial proteins were observed, and the mean IOD value was taken as the IOD value for each uterine specimen. The higher the IOD value, the stronger the positive expression of the proteins.

### ELISA

The enzyme-linked immunosorbent assay (ELISA) of endometrial tissues in the two groups were conducted according to the IL-6 and TGF-β1 assay kits. Absorbance was measured at 450 nm with an enzyme-labeling instrument, and the levels of IL-6 and TGF-β1 were calculated using a standard curve.

### Evaluation of the number of endometrial glands and the fibrotic area in rats

In each H&E-stained section of rat endometrial tissue, four visual fields were randomly selected for analysis under a microscope (50×). The number of endometrial glands in each visual field was counted, and the mean was calculated. Four visual fields (100×) in each Masson’s trichrome-stained section were also randomly selected for microscopic analysis. The area of endometrial interstitial fibrosis, the area of the endometrial stroma, and the glandular density in each visual field were calculated using Image-ProPlus 6.0. The fibrosis ratio was calculated as the mean of the area of endometrial interstitial fibrosis divided by the total area of the endometrial stroma and glands in each visual field.

### Statistical analysis

Statistical analyses were performed using SAS 9.4 (SAS Institute, Cary, NC, USA). Numerical variables were expressed as the mean ± SD. Data were compared using t-tests for count data and one-way analysis of variance (ANOVA) or Fisher’s exact probability method for measurement data. Logistic regression analysis was used to determine the dominant variables for establishing IUA prediction models. A two-sided *P* < 0.05 was considered significant.

## Results

### Clinical baseline characteristics of the IUA and non-IUA groups

In total, 12,297 participants were included in this study including 908 patients with IUA (7.38%; the IUA group) and 11,389 healthy controls (92.62%; the non-IUA group). Clinical baseline parameters of the study population are presented in Table 1. Six cases (0.7%) in the IUA group were HPV positive, whereas none of the 11,389 cases in the non-IUA group had HPV infection. The proportion of patients with a large population of *Lactobacilli* was significantly higher in the IUA group than in the non-IUA group (39.6% vs. 0.2%, respectively; *P* = 0.0000). Compared to the non-IUA group, the IUA group had a higher proportion of cases with abnormal microbiota (56.4% vs. 30%, respectively; *P* = 0.0000) and poorer vaginal cleanliness (*P* = 0.0000). Interestingly, the proportion of cases with fungal infection was significantly higher in the IUA group than in the non-IUA group (18.2% vs. 9.1%, respectively; *P* = 0.0000), as was the proportion of cases with pH > 4.6 (43.2% vs. 3.2%, respectively; *P* = 0.0000). There was no significant difference in the dominant bacteria or acetylglucosaminidase between the two groups (*P* > 0.05).

**Table 1:** Clinical baseline parameters in IUA and non-IUA patients.

Variate	Category	IUA	Non-IUA	*P* value
Age	No. patients	908	11,389	0.0000
	Missing data	0	0	
	Mean ± SD	31.9 ± 5.7	33.4 ± 4.2	
	Median	31	34	
	Interquartile range	28.0–36.0	30.0–37.0	
	Minimum–maximum	15–49	19–40	
HPV	Positive	6 (0.7)	0 (0.0)	0.0000
	Negative	902 (99.3)	11,389 (100.0)	
	Total	908 (100.0)	11,389 (100.0)	
*Lactobacillus*	Large amount	360 (39.6)	20 (0.2)	0.0000
	Medium amount	143 (15.7)	7504 (65.9)	
	Small amount or none	405 (44.6)	3865 (33.9)	
	Total	908 (100.0)	11,389 (100.0)	
Dominant bacteria	Gram-positive	765 (84.3)	9569 (84.0)	0.8877
	Gram-negative	143 (15.7)	1820 (16.0)	
	Total	908 (100.0)	11,389 (100.0)	
Microecology	Abnormal	512 (56.4)	3412 (30.0)	0.0000
	Normal	396 (43.6)	7977 (70.0)	
	Total	908 (100.0)	11,389 (100.0)	
Fungi	Positive	165 (18.2)	1037 (9.1)	0.0000
	Negative	743 (81.8)	10,352 (90.9)	
	Total	908 (100.0)	11,389 (100.0)	
Nugent score	1	206 (22.7)	6098 (53.5)	0.0000
	2	259 (28.5)	1888 (16.6)	
	3	73 (8.0)	3380 (29.7)	
	4 +	370 (40.7)	23 (0.2)	
	Total	908 (100.0)	11,389 (100.0)	
Leukocyte esterase	Positive	121 (13.3)	885 (7.8)	0.0000
	Negative	787 (86.7)	10,504 (92.2)	
	Total	908 (100.0)	11,389 (100.0)	
PH	≤ 4.6	516 (56.8)	10,571 (92.8)	0.0000
	> 4.6	392 (43.2)	818 (7.2)	
	Total	908 (100.0)	11,389 (100.0)	
Acetylglucosaminidase	Positive	73 (8.0)	1036 (9.1)	0.3062
	Negative	835 (92.0)	10,353 (90.9)	
	Total	908 (100.0)	11,389 (100.0)	
Vaginal cleanliness	II	424 (46.7)	9542 (83.8)	0.0000
	II	463 (51.0)	1845 (16.2)	
	IV	21 (2.3)	2 (0.0)	
	Total	908 (100.0)	11,389 (100.0)	

Unless indicated otherwise, data are given as n (%).

*HPV* human papillomavirus, *IUA* intrauterine adhesion.

### Univariate and multivariate logistic regression analysis of factors influencing IUA occurrence

To identify factors that influence IUA, we first conducted univariate logistic regression analysis (Table 2). The amount of *Lactobacillus*, vaginal microecology, fungal infection, Nugent score, leukocyte esterase, H_2_O_2_, pH, and vaginal cleanliness were all identified as variates that may affect the occurrence of IUA. Compared with the non-IUA group, the IUA group was more likely to have vaginal fungal infection [*P* < 0.0001; odds ratio (OR) 0.451, 95% confidence interval (CI): 0.377 to 0.540] and higher vaginal pH (*P* < 0.0001; OR 9.817, 95% CI: 8.456 to 11.399). Multivariate logistic regression analysis further showed that fungal infection, pH, the amount of *Lactobacillus*, Nugent score, H_2_O_2_, and vaginal cleanliness were all significantly correlated with IUA (*P* < 0.05; Table 2).

**Table 2:** Univariate and multivariate logistic regression results of IUA group and non-IUA group.

Variate	Category	Estimate	SE	χ^2^*	*P*-value	OR	95% CI
**Univariate logistic regression analysis**							
Age		–0.0778	0.0078	100.1839	< 0.0001	0.925	0.911–0.939
HPV	Positive	Reference					
	Negative	–15.55	273.4000	0.0032	0.9546	< 0.001	< 0.001– > 999.999
*Lactobacillus*	Large amount	Reference					
	Small amount or none	–5.1452	0.2355	477.3426	< 0.0001	0.006	0.004–0.009
	Medium amount	–6.8498	0.2447	783.8341	< 0.0001	0.001	< 0.001–0.002
Dominant bacteria	Gram-positive	Reference					
	Gram-negative	–0.0173	0.0946	0.0332	0.8553	0.983	0.817–1.183
Microecology	Abnormal	Reference					
	Normal	–1.1062	0.0700	249.9123	< 0.0001	0.331	0.288–0.379
Fungi	Positive	Reference					
	Negative	–0.7962	0.0920	74.8759	< 0.0001	0.451	0.377–0.540
Nugent score	1	Reference					
	2	1.4014	0.0970	208.7259	< 0.0001	4.061	3.358–4.911
	3	–0.4473	0.1379	10.5248	0.0012	0.639	0.488–0.838
	≥ 4	6.1656	0.2263	742.6268	< 0.0001	476.091	305.566–741.780
Leukocyte esterase	Positive	Reference					
	Negative	–0.6015	0.1037	33.6249	< 0.0001	0.548	0.447–0.672
H_2_O_2_	Positive	Reference					
	Negative	5.389	0.1606	1126.5878	< 0.0001	218.987	159.865–299.975
pH	≤ 4.6	Reference					
	> 4.6	2.2842	0.0762	898.6103	< 0.0001	9.817	8.456–11.399
Acetylglucosaminidase	Positive	Reference					
	Negative	0.135	0.1263	1.1428	0.2851	1.145	0.894–1.466
Vaginal cleanliness	II	Reference					
	III	1.7312	0.0719	580.2604	< 0.0001	5.648	4.906–6.502
	IV	5.4644	0.7415	54.3125	< 0.0001	236.143	55.212– > 999.999
**Multivariate logistic regression analysis**							
Intercept		0.7979	0.9597	0.6913	0.4057	**–**	**–**
Age		–0.0716	0.021	11.6276	0.0006	0.931	0.893–0.97
*Lactobacillus*	Small amount or none	–4.7647	0.6564	52.693	< 0.0001	0.009	0.002–0.031
	Medium amount	–3.2884	0.4962	43.9173	< 0.0001	0.037	0.014–0.099
Fungi	Negative	1.0186	0.4033	6.3778	0.0116	2.769	1.256–6.105
Nugent score	2	4.1099	0.4355	89.059	< 0.0001	60.94	25.954–143.088
	3	2.3001	0.5019	21.0056	< 0.0001	9.975	3.73–26.674
	≥ 4	10.7942	0.7558	203.9526	< 0.0001	> 999.999	> 999.999– > 999.999
Leukocyte esterase	Negative	1.3537	0.4091	10.9483	0.0009	3.872	1.736–8.633
H_2_O_2_	Negative	5.6609	0.3479	264.7112	< 0.0001	287.404	145.321–568.402
pH > 4.6	> 4.6	2.7971	0.4012	48.6126	< 0.0001	16.397	7.469–35.994
Vaginal cleanliness	III	–2.166	0.4689	21.3405	< 0.0001	0.115	0.046–0.287
	IV	6.9129	0.953	52.6171	< 0.0001	> 999.999	155.253– > 999.999

*CI* confidence interval, *HPV* human papillomavirus, *IUA* intrauterine adhesion, *OR* odds ratio.

*Chi-squared test for the entire group.

### General appearance of uterine samples after *C. albicans* perfusion in rats

**Figure 1:**
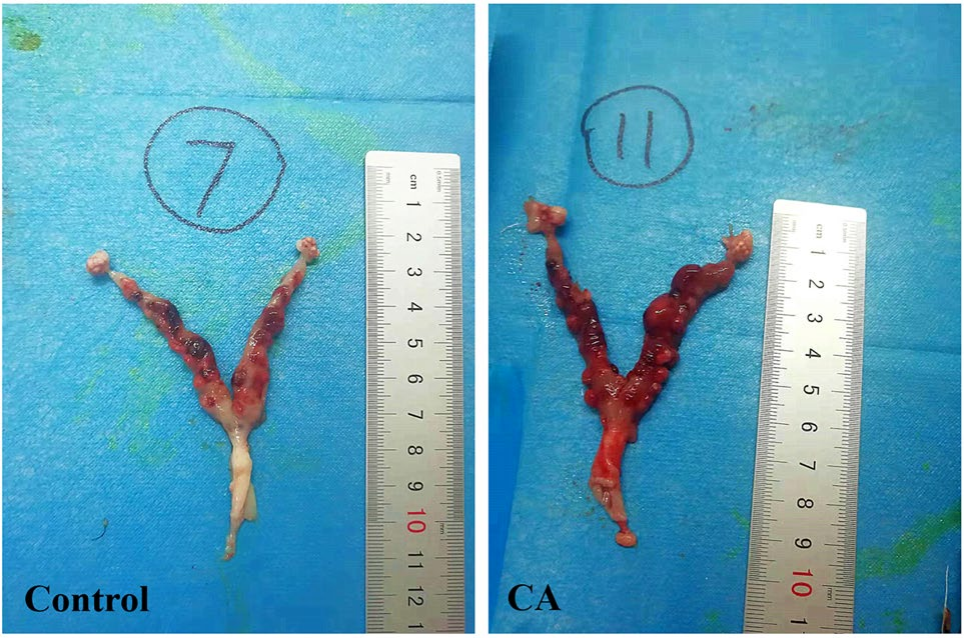
General appearance of uterine specimens on day 3 after *Candida albicans* (CA) injection into the reproductive tract of rats (right) compared with the control group (left). CA, *Candida albicans* group.

The uterine samples from the *C. albicans* and control groups were not fully contracted on the 3rd day postoperatively. However, the inflammatory edema in the *C. albicans* group was slightly more severe than that in the control group (Fig. 1).

### Changes in inflammation and fibrosis of the reproductive tract after *C. albicans* perfusion

**Figure 2:**
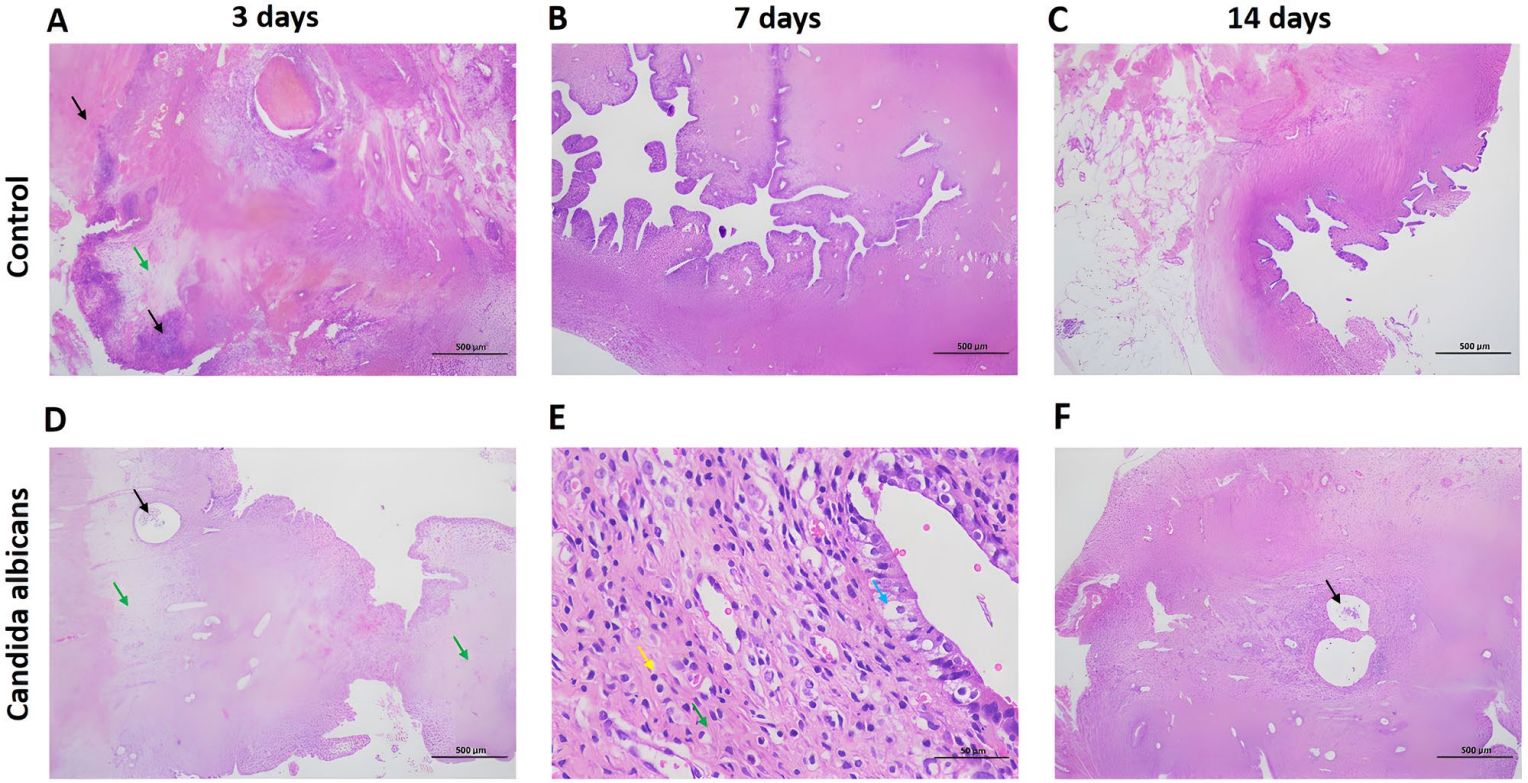
H&E staining of rat endometrial tissues on postoperative days 3, 7, and 14 in the *Candida albicans* and control groups. (**A**) On day 3, H&E staining results in the control group show suppurative necrosis (black arrows) and local edema (green arrow). (**B,C**) H&E staining results in the control group on the day 7 and 14. (**D**) On day 3, H&E staining results in the *C. albicans* group show neutrophils (black arrow) and lamina propria edema (green arrows). (**E**) On day 7, H&E staining results in the *C. albicans* group show local uterine epithelial cytoplasmic vacuolation (blue arrow), a small number of lymphocytes (yellow arrow), and more collagen fiber hyperplasia (green arrow) in the lamina propria. (**F**) On day 14, H&E staining results in the *C. albicans* group show inflammatory exfoliation and deletion of the uterine epithelium (black arrow). *H&E* hematoxylin and eosin.

In the control group, on postoperative day 3, large areas of necrosis, coagulative necrosis, suppurative necrosis, bleeding foci, and local edema were observed. A small number of glandular structures and more collagen fibers could also be seen in local tissues (Fig. 2A). On postoperative day 7, a small number of glands, a small number of neutrophils in the lamina propria, and more collagen fiber hyperplasia were observed (Fig. 2B). On day 14 after the procedure, there were no obvious glandular structures, but we did observe a small amount of cytoplasmic loss and nuclear pyknosis in the uterine luminal epithelium, scattered neutrophils in the lamina propria, and more collagen fibers (Fig. 2C).

In the *C. albicans* group, we observed a small number of glandular structures in local tissues, dilated glands, and neutrophils in some glandular cavities on postoperative day 3, as well as more neutrophil infiltration in the lamina propria, necrotic foci and eosinophilia in tissues, lamina propria edema, and multiple local hemorrhagic foci (Fig. 2D). On postoperative day 7, we observed a small number of glandular structures in the lamina propria, necrosis and nuclear fragmentation in some glandular epithelial cells, local uterine epithelial cytoplasmic vacuolation, a small number of lymphocytes, and more collagen fiber hyperplasia in the lamina propria (Fig. 2E). Furthermore, a hematoma had formed in the local tissue, which was surrounded by connective tissue, forming a capsule, and there was phagocytosis of hemosiderin-laden macrophages in the outer layer (Fig. 2E). On day 14 after the procedure, we observed inflammatory exfoliation and deletion of the uterine epithelium, exfoliated epithelial cells, necrotic tissue fragments, neutrophils, hyperemia, dilatation of the subepithelial capillaries, no obvious glandular structures, more inflammatory cell infiltration, and local fibroblast hyperplasia in the lamina propria (Fig. 2F).

### Changes in inflammation and fibrosis of the reproductive tract after *C. albicans* perfusion

**Figure 3:**
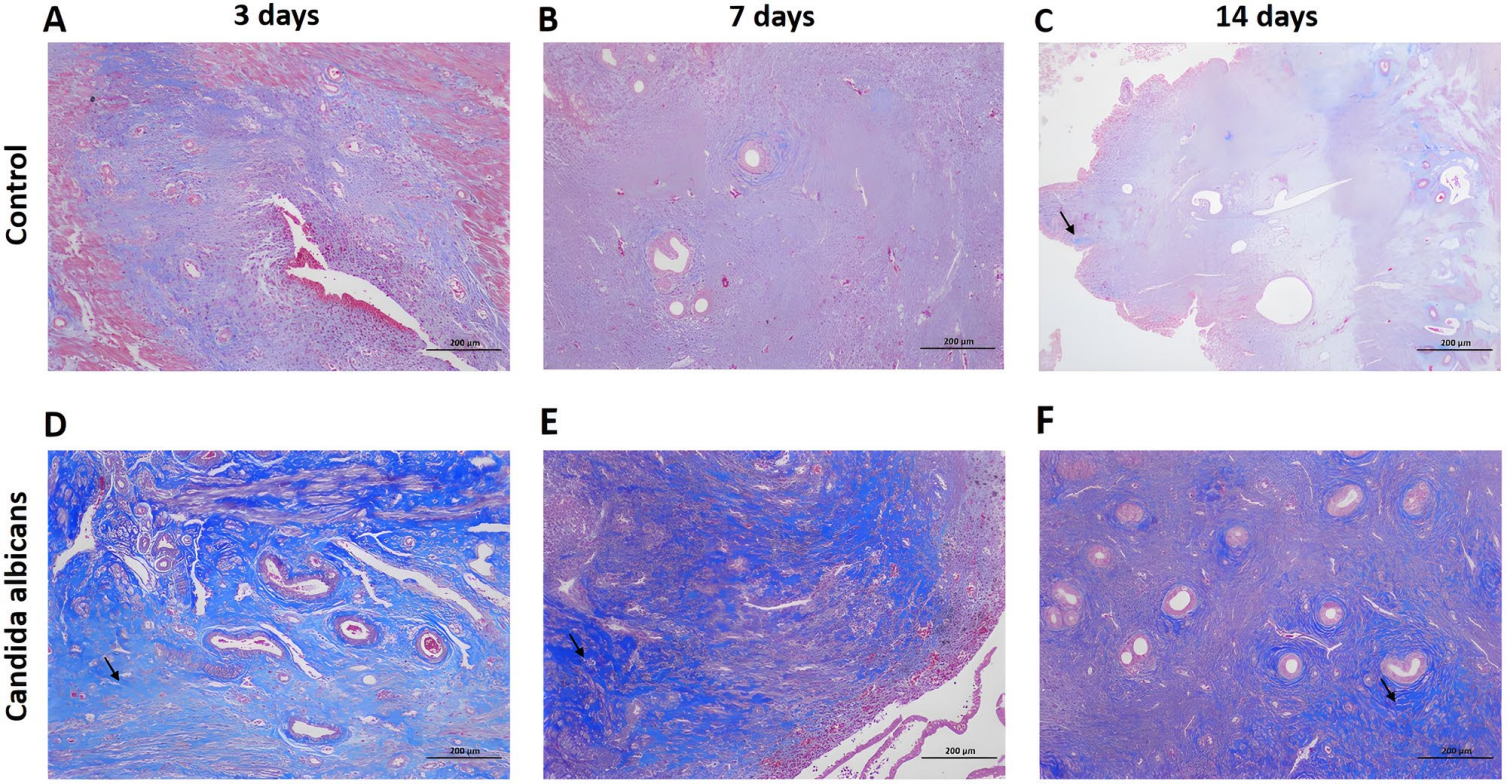
Masson’s trichrome staining of rat endometrial tissues in the control and *C. albicans* groups on postoperative days 3, 7, and 14. (**A,B**) Masson trichrome staining results in the control group on the day 3 and 7. (**C**) On day 14, staining results in the control group show slight, collagen fiber proliferation (black arrow). (**D**) On day 3, staining results in the *C. albicans* group show severe collagen fiber hyperplasia (black arrow). (**E**) On day 7, staining results in the *C. albicans* group show extremely severe hyperplasia of the collagen fibers, with some of the fibers forming strips (black arrow). (**F**) On day 14, staining results in the *C. albicans* group show severe collagen fiber proliferation can be seen, with some of the fibers forming strips.

In the control group, mild to moderate collagen fiber hyperplasia was observed on day 3 (Fig. 3A), and there was slight collagen fiber proliferation on days 7 (Fig. 3B) and 14 (Fig. 3C).

In the *C. albicans* group, severe collagen fiber hyperplasia was observed on postoperative day 3 (Fig. 3D). On day 7, extremely severe collagen fiber hyperplasia was observed, with some of the fibers forming strips (Fig. 3E). On day 14, there was severe collagen fiber proliferation, with some of the fibers forming strips (Fig. 3F).

The mean number of endometrial glands in the control and *C. albicans* groups was 36.230 ± 20.630 and 19.320 ± 12.780 /mm^2^, respectively. The endometrial gland density in the *C. albicans* group was significantly lower than that in the control group (*P* < 0.001, Table 3). On postoperative days 3, 7, and 14, the ratio of the endometrial fibrosis area to the the total endometrial area in the control group was 0.128 ± 0.058, 0.106 ± 0.038, and 0.069 ± 0.024, respectively; in the *C. albicans* group, it was 0.211 ± 0.142, 0.371 ± 0.059, and 0.413 ± 0.131, respectively. These results show that the degree of endometrial fibrosis stimulated by *C. albicans* in the IUA model rats was significantly greater than that in the control group at three different time points (postoperative days 3, 7, and 14) (*P* < 0.001; Table 3).

**Table 3:** Ratio of endometrial fibrosis area to total area and endometrial gland density in the control and *Candida albicans* groups.

	Control group	*Candida albicans* group	*P-*value
**Endometrial glands (1/m**^**2**^**)**	36.230 ± 20.630	19.320 ± 12.780	< 0.001
**Endometrial fibrosis area/total area**			
POD 3	0.128 ± 0.058	0.211 ± 0.142	< 0.001
POD 7	0.106 ± 0.038	0.371 ± 0.059	< 0.001
POD 14	0.069 ± 0.024	0.413 ± 0.131	< 0.001

Unless indicated otherwise, data are given as the mean ± SD.

*POD* postoperative day.

### Expression of IL-6, TGF-β1, Smad 2, COLl, ER, and PR in the endometrium of IUA model rats

**Figure 4:**
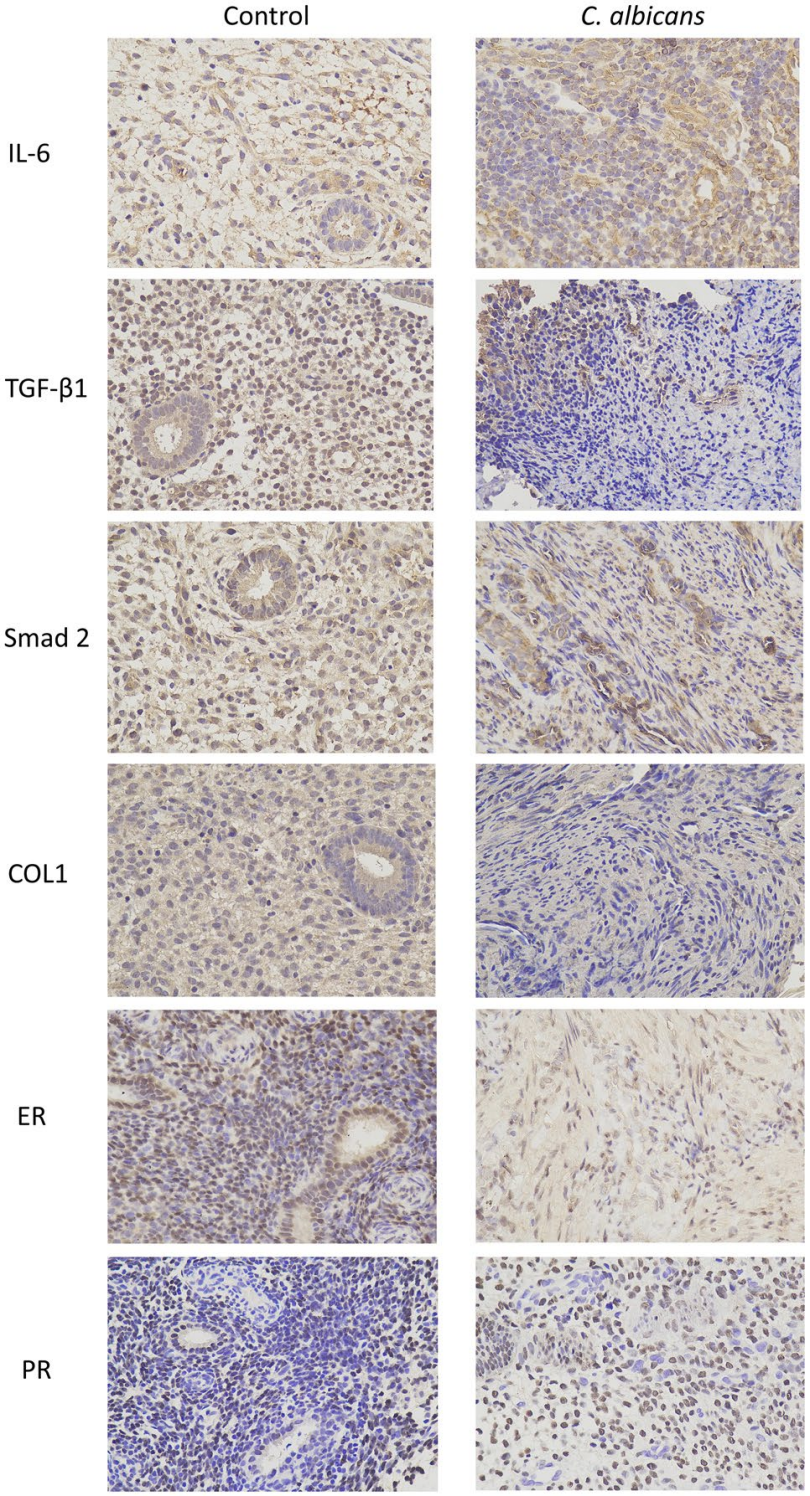
Immunohistochemical staining of IL-6, TGF-β1, Smad 2, COL1, ER, and PR in the *C. albicans* and control groups (×400). *IL-6* interleukin-6, *TGF-β1* transforming growth factor-β1,* ER* estrogen receptor, *PR* progesterone receptor.

The expression levels of the IL-6, TGF-β1, Smad 2, COLl, ER and PR proteins in the endometrium, as assessed by IHC, were significantly higher in the *C. albicans* group than in the control group, whereas the levels of ER and PR were significantly lower in the *C. albicans* group than in the control group (Fig. 4, Table 4). The expression levels of IL-6 and TGF-β1 in the endometrium, as detected by ELISA, were found to be significantly higher in the *C. albicans* group than in the control group (Table 5).

**Table 4:** Expression of IL-6, TGF-β1, COL1, ER, and PR in the *C. albicans* and control groups, as determined by IHC.

	IL-6	TGF-β1	Smad 2	coll 1	ER	PR
Control	0.015	0.017	0.024	0.013	0.020	0.021
*C. albicans*	0.025*	0.026*	0.052**	0.031*	0.015*	0.010**

*ER* estrogen receptor, *IL-6* interleukin-6, *PR* progesterone receptor, *TGF-β1* transforming growth factor-β1, *IHC* immunohistochemistry.

*P < 0.05, **P < 0.01 compared with control.

**Table 5:** Mean concentration of IL-6 and TGF-β1 in the endometrium of rats in the *C. albicans* and control groups, as determined by ELISA.

	IL-6 (pg/mL)	TGF-β1 (pg/mL)
Control	23.659	168.373
*C. albicans*	40.643**	233.815*

*ELISA* enzyme-linked immunosorbent assay, *IL-6* interleukin-6, *TGF-β1* transforming growth factor-β1.

*P < 0.05, **P < 0.01 compared with control.

## Discussion

The reproductive tract microbiome is an extensive and diverse system of pathogenic and non-pathogenic microorganisms that play an important role in female reproductive health^40,41^. In one study, endometrial samples from 46 patients with IUA and 21 infertile patients without IUA were analyzed. The results showed that the proportions of *Klebsiella*, *Shewanella*, and *Lactobacillus* at the genus level were higher in patients with IUA than those without IUA, whereas the proportion of *Acinetobacter* was significantly lower in patients with IUA^42^. Liu et al.^43^ collected samples of lower genital tract secretions from 50 patients with IUA and 30 healthy women for microbiological evaluation. In their study, patients with IUA had a significantly lower percentage of *Firmicutes* and a higher percentage of *Actinobacteria* at the phylum level than did women in the healthy vaginal secretion group^43^. At the genus level, nearly 50% of patients with IUA were found to have markedly reduced levels of the probiotic *Lactobacillus*, accompanied by an excess of pathogenic *Gardnerella* and *Prevotella*^43^. Their study also pointed out that patients with IUA lacked some normal bacteria, which may impair the endometrium’s repair functions^43^.

Through a retrospective analysis of a large clinical sample, we found that the fungal infection rate in patients with IUA was higher than that in healthy women and was accompanied by an increased vaginal pH, and fungal infection was further revealed to be an independent risk factor for IUA. It was also found that vaginal fungal infection in patients with IUA occurred simultaneously with an increase in *Lactobacillus*. This finding is in contrast with the results of a previous study which compared the vaginal microbiota of 50 patients with IUA and 30 healthy women and found that the amount of *Lactobacillus* was significantly decreased in approximately 50% of the IUA group^43^. It has been confirmed that vaginal fungal infections can be accompanied by an increase in pH^44^. We speculate that the reason for this increase may be that fungal infection elevates the pH of the vagina, which cannot be offset by *Lactobacillus*. It remains unclear whether this serves as a compensatory mechanism by which the vaginal microecosystem in patients with IUA can maintain a normal vaginal pH so as to resist fungal infection, and whether the increase in vaginal *Lactobacillus* in patients with IUA results from fungal infection. Further investigation is required to address these issues.

In recent years, fungal vaginitis has been considered to be an immunopathological reaction^45^. A study has shown that vaginal epithelial cells can distinguish colonized yeast from invasive hyphae by activating pathways such as the oxidative stress pathway^46^. Furthermore, invasive hyphae have been associated with cell damage^46^. It has also been shown that *C. albicans* with mycelial deletion strains can colonize as well as, or even better than, wild-type strains but cannot induce immunopathological markers or mucosal damage^46^. Fungal infection of the reproductive tract is not necessarily accompanied by clinical symptoms^47^. These results suggest that in fungal vaginitis, the transition from spores to hyphae or downstream mycelium-related effectors may be the key to tissue damage and subsequent immunopathological processes^47^.

It has been reported that *C. albicans* can cause a strong proinflammatory response, upregulate the expression of cytokines, and activate the nuclear factor (NF)-κB signaling pathway^48^. The increase in inflammatory cytokines actively promotes the occurrence and development of IUA^49^. The NF-κB pathway plays an important role in the production of cytokines and cell survival, and is an important part of the immune response, having been found to actively promote the pathogenesis of many inflammatory diseases^50^. Researchers have found significantly increased mRNA and protein levels of NF-κB in patients with IUA and in IUA rat models^51^. Through IHC studies, activation of the NF-κB signal in IUA has been further confirmed, and a close relationship has been detected between the NF-κB signaling pathway and the pathogenesis of IUA, as well as cytokines involved in the pathogenesis of IUA, such as TGF-β1, tumor necrosis factor (TNF)-α, and IL-6^51^. Research has also shown that the activation and expression of the NF-κB signaling pathway in IUA tissue plays an important role in the pathogenesis of the condition^51^.

IL-6 can promote fibrosis and activate the TGF-β/Smad pathway. The IL-6 receptor (IL-6R) is usually membrane bound, although it also exists in a soluble form (sIL-6R). When IL-6 binds to sIL-6R, a complex is formed which activates membrane-bound glycoprotein 130. This complex is constitutively expressed in most cell types. IL-6 binding to SIL-6R also leads to the activation of signal transducers and activators of transcription (STAT) signaling pathways, known as IL-6 trans-signals^48^. Importantly, unlike other soluble cytokine receptors, sIL-6R does not antagonize the activity of IL-6,instead, it has the opposite effect. While sIL-6R can be formed by limited proteolysis of membrane-bound receptors, it can also be secreted directly from cells through selective mRNA splicing^29^. Fibrotic diseases are characterized by the activation of fibroblasts. In the present study, we found that fibroblast activation was attributable to increases in the levels of the inflammatory cytokines TNF-α and IL-6. We also found that IL-6 upregulated the factors related to the TGF-β signaling pathway.

As a key mediator of tissue and organ fibrosis, TGF-β1 has a significant effect on fibroblast proliferation and the production of extracellular matrix in vitro^52^. A major process in TGF-β1 signal transduction is the recognition of Smad proteins^22^. Receptor-Smads (R-Smads,including Smad 2 and Smad 3) are the main downstream effectors of the TGF-β signaling pathway^53^. Considering the key role of IL-6 in the production of TGF-β1, we speculate that IL-6 may activate the Smad signaling pathway. After *C. albicans* perfusion, we observed that the expression of Smad 2 in endometrial sections was upregulated relative to that in the control group, which indicates that the activation of Smad 2 may be one of the intracellular mechanisms of IL-6-mediated endometrial fibrosis. Recent data have provided new evidence that the Smad protein is mainly activated by the classical TGF-β1 trigger mechanism^22^. Our results also show that IL-6-induced Smad activation was mediated by TGF-β1 in our rat fibrosis model.

The current study has several limitations that should be noted. First, we identified a problem based on retrospective clinical study of a large sample, and then conducted preliminary verification through an animal experiment. We identified an *C. albicans*-TGF-β/Smad-IL-6 axis, but did not confirm its regulatory relationship through further experiments. The *C. albicans* was migrated from vagina to uterine cavity, but not colonized in the uterine cavity directly. Therefore, inflammation in the uterus may be affected by inflammation in the vagina, and the increase in inflammatory cytokines might be associated to the occurrence and development of IUA. A more effective and valuable animal model of IUA needs further exploration. Despite these limitations, this is the first study to explore the pathogenesis of IUA from the perspective of reproductive tract fungal infection. Our findings provide a new insight for understanding the pathogenesis of IUA and a new direction for the treatment and prevention of this condition. In future research, the regulation of *C. albicans*-TGF-β/Smad-IL-6 axis needs to be verified in vivo or in vitro.

In conclusion, this study has found that vaginal microecological disorders exist in patients with IUA, and that vaginal fungal infection may be one of the risk factors for IUA. *C. albicans* may cause a strong inflammatory response involving IL-6 upregulation and COL1 deposition, which aggravates endometrial fibrosis. *C. albicans* infection may therefore be related to IUA.

## Data Availability

The datasets used and/or analysed during the current study available from the corresponding author on reasonable request.
